# Decreased Expression of Soluble Epoxide Hydrolase Suppresses Murine Choroidal Neovascularization

**DOI:** 10.3390/ijms232415595

**Published:** 2022-12-09

**Authors:** Bomina Park, Sheik Pran Babu Sardar Pasha, Kamakshi L. Sishtla, Gabriella D. Hartman, Xiaoping Qi, Michael E. Boulton, Timothy W. Corson

**Affiliations:** 1 Eugene and Marilyn Glick Eye Institute, Department of Ophthalmology, Indiana University School of Medicine, Indianapolis, IN 46202, USA; 2 Department of Pharmacology and Toxicology, Indiana University School of Medicine, Indianapolis, IN 46202, USA; 3 Stark Neurosciences Research Institute, Indiana University School of Medicine, Indianapolis, IN 46202, USA; 4 Department of Ophthalmology and Visual Sciences, University of Alabama at Birmingham, Birmingham, AL 35294, USA; 5 Department of Biochemistry and Molecular Biology, Indiana University School of Medicine, Indianapolis, IN 46202, USA

**Keywords:** adenoassociated viral vector, choroidal neovascularization, neovascular age-related macular degeneration, RNAscope, soluble epoxide hydrolase

## Abstract

Neovascular or “wet” age-related macular degeneration (nAMD) is a leading cause of blindness among older adults. Choroidal neovascularization (CNV) is a major pathological feature of nAMD, in which abnormal new blood vessel growth from the choroid leads to irreversible vision loss. There is a critical need to develop novel therapeutic strategies to address limitations of the current anti-vascular endothelial growth factor biologics. Previously, we identified soluble epoxide hydrolase (sEH) as a possible therapeutic target for CNV through a forward chemical genetic approach. The purpose of this study was to validate sEH as a target by examining retinal expression of sEH protein and mRNA by immunohistochemistry and RNAscope in situ hybridization, respectively, and to assess the efficacy of an adeno-associated virus (AAV) vector designed to knock down the sEH gene, *Ephx2*, in the murine laser-induced (L-) CNV model. nAMD patient postmortem eye tissue and murine L-CNV showed overexpression of sEH in photoreceptors and retinal pigment epithelial cells. *Ephx2* knockdown significantly reduced CNV and normalized mRNA expression levels of CNV-related inflammatory markers. Thus, this study further establishes sEH as a promising therapeutic target against CNV associated with nAMD.

## 1. Introduction

Age-related macular degeneration (AMD) is a retinal neurodegenerative disease that is the leading cause of blindness in older adults in the industrialized world [1]. Currently, 11 million people in the United States and 187 million people globally are estimated to have AMD [2]. The advanced form of the disease, neovascular AMD (nAMD), which accounts for 90% of the blindness resulting from AMD [3], is distinguished by choroidal neovascularization (CNV), aberrant blood vessel growth from the choroid into the retinal pigment epithelium (RPE) and neuroretina [4]. Leakage of these new blood vessels causes hemorrhage, retinal pathology, and eventual fibrotic scarring, with rapid, permanent central vision loss [5]. CNV involves abnormal tissue invasion of endothelial cells from the choroid and inflammatory cells, and both angiogenesis and inflammation contribute to the pathogenesis [6]. However, current approved therapy for nAMD is limited to targeting vascular endothelial growth factor (VEGF) angiogenic pathways (and recently also angiopoietin signaling [7]) with biologics. Although these biologics have improved outcomes, these treatments are complicated by the emergence of resistance and refractory responses [8]. Therefore, development of novel therapeutic strategies is desired to address unmet needs in treatment.

We previously used a forward chemical genetics approach with a lead antiangiogenic small molecule, SH-11037, and identified soluble epoxide hydrolase (sEH) as a target for inhibiting CNV [9]. sEH, encoded by *EPHX2*, is a bifunctional enzyme with a C-terminal hydrolase function that is responsible for the hydrolysis of polyunsaturated fatty acid (PUFA)-derived epoxy fatty acids (EpFA) which have distinct bioactivities in pathological angiogenesis and inflammation [10]. It also has less well-characterized N-terminal lipid phosphatase activity. Inhibition of sEH hydrolase activity stabilizes endogenous EpFAs, such as epoxydocosapentaenoic acids (EDP) from omega-3 docosahexaenoic acid (DHA, ω-3). EDPs are known as pro-resolving lipid mediators that promote resolution of inflammation. The functions of EpFAs, which are potentiated by sEH inhibition, have been characterized in neurodegeneration, wound healing, vascular remodeling, and angiogenesis [11,12,13,14,15,16,17,18,19,20].

Although we had previously demonstrated that sEH is upregulated in L-CNV [9], a knowledge gap lies in the precise retinal cellular expression of sEH. To address this, we assessed expression patterns of not only sEH protein (by immunohistochemistry), but also its mRNA (by RNAscope in situ hybridization) in disease-relevant tissues. We then knocked down *Ephx2* in the murine L-CNV model and evaluated disease severity and changes in CNV-related gene expression.

## 2. Results

### 2.1. IHC Evaluation of sEH Expression in Human Retinas

**Figure 1:**
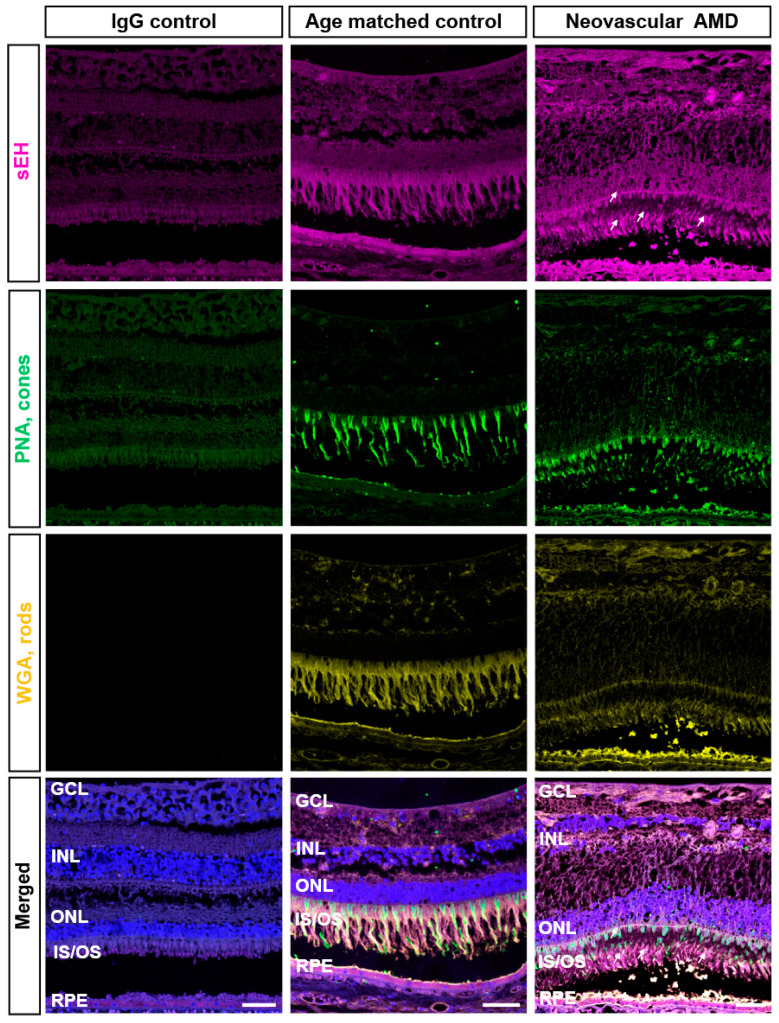
Colocalization of sEH with photoreceptors in human retinas from neovascular AMD subjects. Representative images of central retinal sections from neovascular AMD (81 year-old [y.o.], male) and age-matched control eyes (82 y.o., male) stained with DAPI (blue), sEH (magenta), peanut agglutinin (PNA; green), or wheat germ agglutinin (WGA; yellow). IgG control is from a normal control eye (34 y.o., male) with preimmune serum instead of primary antibody. Labeling of retinal tissues with PNA and WGA for rods and cones shows colocalization of sEH in photoreceptors. In nAMD, sEH staining is strongly associated with the outer segments of cones and is enhanced compared to age-matched control (indicated by arrows). Scale bars: 50 μm. Representative images from *n* = 2 control and *n* = 3 nAMD subjects; see also Figure S1.

**Figure 2:**
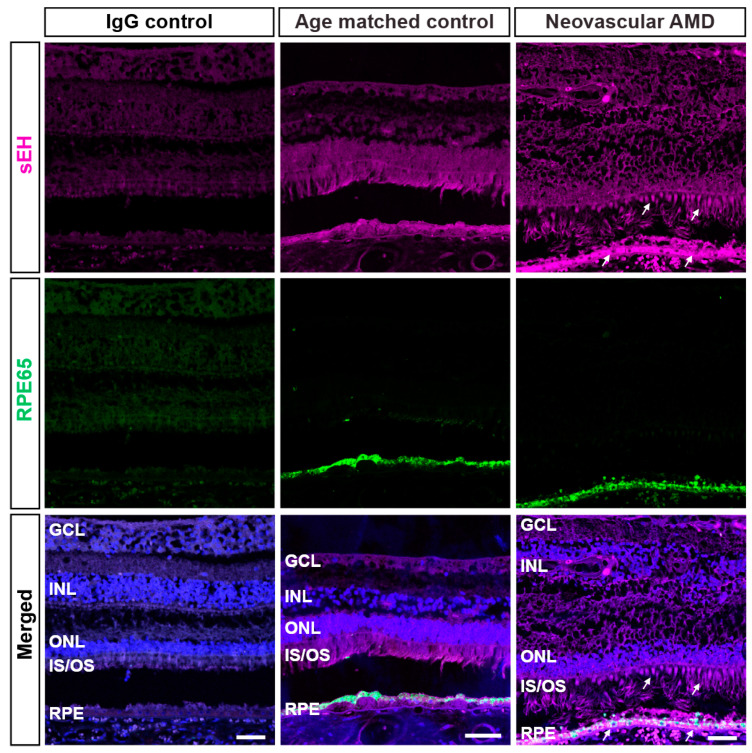
Colocalization of sEH with RPE in human retinas from neovascular AMD subjects. Representative images of central retinal sections from neovascular AMD (81 y.o., male) and age-matched control eyes (82 y.o., male) stained with DAPI (blue)**,** sEH (magenta), or RPE65 (green). IgG control is from a normal control eye (34 y.o., male) with preimmune serum instead of primary antibody. Immunohistochemistry using antibodies against sEH and RPE65 shows positive staining of sEH in RPE cells with more intense expression in neovascular AMD retinal tissue (indicated by arrows). Scale bars: 50 μm. Representative images from *n* = 2 control and *n* = 3 nAMD subjects; see also Figure S1.

We aimed to build on our previous findings that sEH is overexpressed in the vasculature and outer retinas of human nAMD [9]. To further define the localization of sEH, we immunostained for sEH and other markers of retinal cell types: peanut agglutinin (PNA) for cone photoreceptor cells, wheat germ agglutinin (WGA) for rod photoreceptor cells, and RPE65 for RPE cells. PNA (green) distinctively identified cone matrix sheaths and WGA (yellow) labeled outer segments of rods (Figure 1). The expression of sEH was observed throughout the retina, but greater sEH expression was observed in photoreceptors and RPE cells that could be undergoing degenerative changes in nAMD. sEH staining was associated with inner and outer segments of photoreceptor cells in nAMD retinas (Figure 1). Co-immunostaining of RPE65 and sEH revealed that sEH overexpression in nAMD was also associated with RPE65-positive RPE cells, particularly in the apical RPE (Figure 2). This observation was confirmed by further examples of sEH staining in human retinas (Figure S1). While this expression pattern is consistent with our immunohistochemistry data on L-CNV murine retinas [9], the results are distinguished from other immunohistochemistry data in the literature in which sEH was predominantly expressed by Müller glial cells in murine retinas with diabetic retinopathy [21,22]. Thus, this presented a rationale to pursue the RNAscope in situ hybridization (ISH) method to provide complementary information regarding precise discrimination of the target expression in retinas undergoing CNV.

### 2.2. RNAscope ISH Evaluation of EPHX2/Ephx2 Expression in Human and Murine Retinas

**Figure 3:**
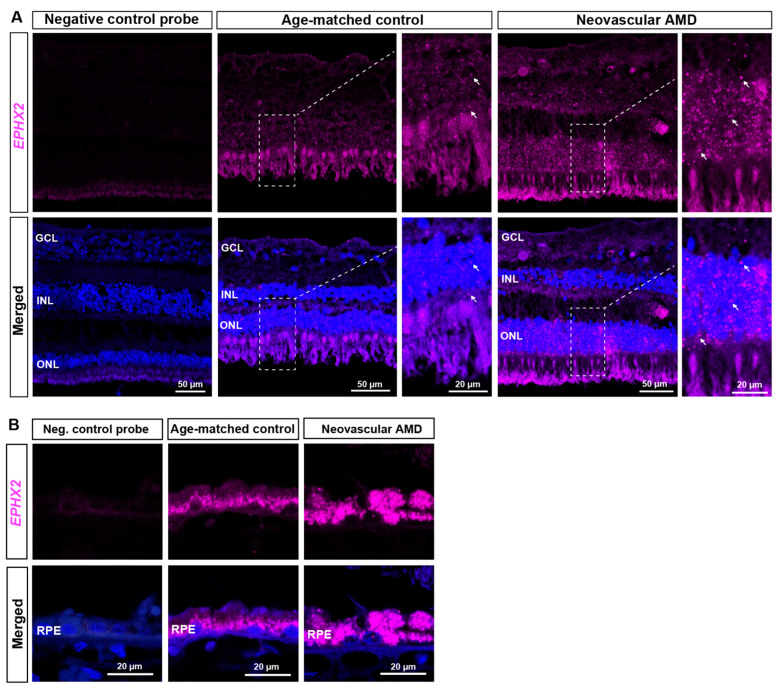
*EPHX2* mRNA is highly expressed in the retinal pigment epithelium (RPE) of AMD eyes. Representative images of retinal sections from neovascular AMD (72 y.o., female) and age-matched control eyes (79 y.o., female). *EPHX2* encoding sEH is in magenta, and nuclei (DAPI) are in blue. (**A**) *EPHX2* mRNA is detected across the retina, including vasculature and photoreceptor cell bodies. Insets show higher magnification images of the indicated areas. *EPHX2* mRNA is highly expressed in the photoreceptor cell bodies in the ONL (white arrows). (**B**) Higher magnification images of the RPE in different areas of the same specimens. *EPHX2* mRNA is highly expressed in RPE. In neovascular AMD, *EPHX2* mRNA signal is increased in the INL, ONL and apical RPE. Bright fluorescent puncta represent positive mRNA signal. Scale bars as indicated. Representative images from *n* = 2 control and *n* = 3 nAMD subjects; see also Figure S2.

Fluorescent RNAscope ISH was performed to achieve single cell resolution for *EPHX2/Ephx2* mRNA detection in human nAMD and murine L-CNV retinas. The *EPHX2* mRNA expression pattern was consistent with what we observed at the protein level through immunohistochemistry. *EPHX2* was broadly localized in multiple cellular layers (Figure 3A), but prominently in photoreceptor cell bodies in the outer nuclear layer (ONL) (Figure 3A and Figure S2), and in RPE (Figure 3B and Figure S2). *EPHX2* mRNA was markedly increased in nAMD eyes, throughout the retina but most prominently in the RPE (Figure 3A,B).

**Figure 4:**
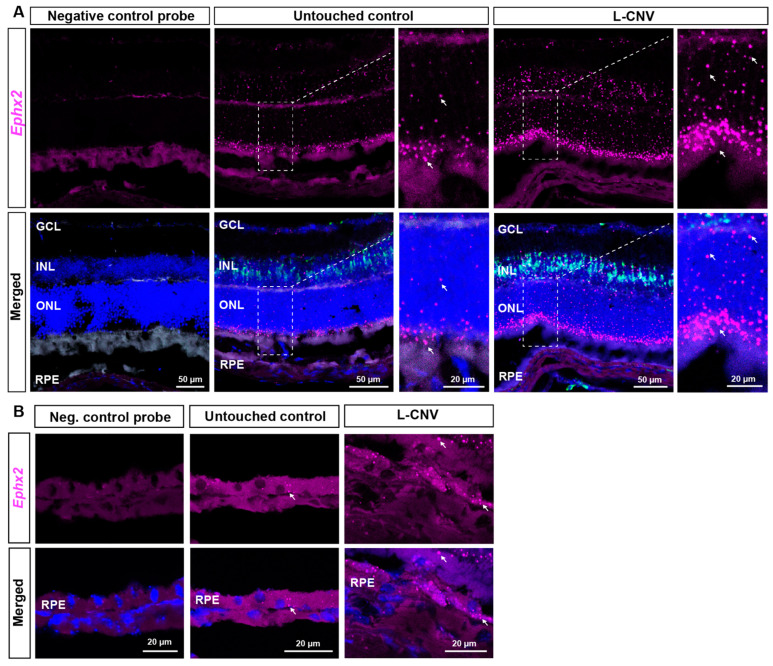
sEH mRNA expression is increased in the photoreceptors and retinal pigment epithelium (RPE) of mouse L-CNV eyes. Representative images of retinal sections near L-CNV lesions from eyes of murine L-CNV 3 days post-laser (labeled as L-CNV in horizontal box) and untouched control eyes. *Ephx2* mRNA encoding murine sEH is in magenta, *Apoe* mRNA (glial cell marker) is in green, and DAPI is in blue. (**A**) *Ephx2* mRNA is detected throughout all cellular layers of the retina, but its signal is most prominently increased in the INL and ONL of the photoreceptor cells in L-CNV (white arrows). *Ephx2* is detected in the INL where *Apoe* was also localized in glial cells. *Ephx2* mRNA is present at high levels in the ONL, as shown in the inset images of the same sections taken at higher magnification. (**B**) *Ephx2* mRNA is expressed at low levels in the RPE of the untouched control, whereas its expression is enhanced in the RPE of the L-CNV eyes (white arrows). Bright fluorescent puncta represent positive RNA signal. Scale bars as indicated. Representative images from *n* = 3 control and *n* = 3 L-CNV mice.

Findings in murine retinas were similar, where *Ephx2* mRNA was localized in photoreceptor cell bodies in the ONL, but also in the inner nuclear layer (INL) and at a low level in RPE of untouched eyes (Figure 4). In L-CNV, there was a notable increase in *Ephx2* mRNA in the photoreceptors and INL, comparable to that seen in human nAMD. However, the increase in *Ephx2* signals in RPE was more modest in the acute murine L-CNV model than in human nAMD eyes (Figure 3B). sEH protein expression has previously been reported in murine Müller glia [21,22]. Apolipoprotein E (*Apoe*) is synthesized in the retina by Müller glial cells [23]; therefore, *Apoe* mRNA was previously used as a target in RNAscope assays to identify Müller glia [24]. We confirmed that *Ephx2* is detected in the INL where *Apoe,* expressed by Müller glia, was localized (Figure 4A). This is consistent with what others reported in developing retina and murine retinas with diabetic retinopathy [21,22]. Overall, however, *Ephx2* overexpression is more prominent in photoreceptors and RPE in murine L-CNV (Figure 4A,B).

### 2.3. In Vivo Transduction of AAV8-Ephx2 shRNA

**Figure 5:**
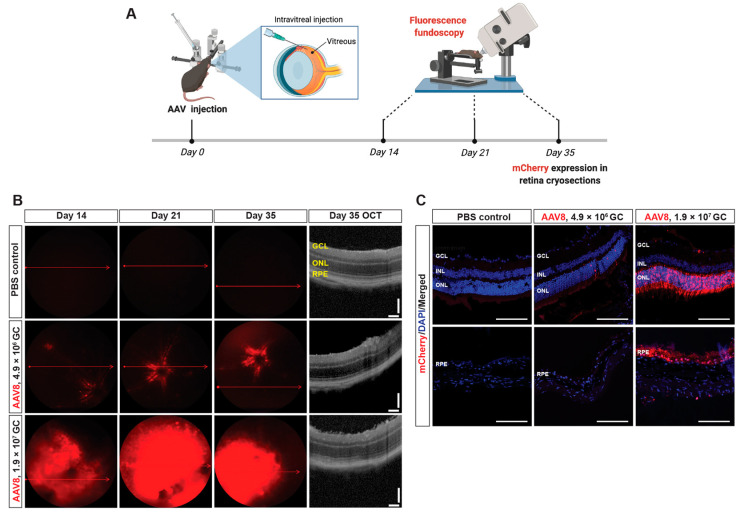
In vivo transduction of AAV8 in murine retina. (**A**) Study design and experimental timeline used to assess in vivo efficacy of AAV8-*Ephx2* shRNA. Noninvasive fluorescence fundoscopy was used to assess mCherry expression in vivo on day 14, 21, and 35 post-injection, followed by cryosection imaging ex vivo. (**B**) Time-dependent and viral dose-dependent transduction efficiency. Fluorescence fundoscopy showing in vivo mCherry expression in wide view image of retina (red lines indicating OCT scan positions) and corresponding OCT images on day 35 showing no substantial changes in retinal structure. (**C**) AAV transduction visualized in retinal sections five weeks after injection. Retinal sections showing mCherry positive cells (red) in ONL and RPE. Nuclei (DAPI) are blue. GC, genome copies. Scale bars = 100 µm.

Given the photoreceptor and RPE-localized overexpression of *Ephx2* in L-CNV, we chose an AAV8 vector to target disease-relevant cells for delivering a short hairpin RNA (shRNA) to knock down *Ephx2*, since AAV8 is well known to transduce photoreceptor and RPE cells [25]. To determine the viral dose and experimental timeline for optimal transduction efficiency, AAV8 vectors encoding U6 promoter-driven *Ephx2* shRNA and a CMV promoter-driven mCherry at low or high titer (4.9 × 10^6^ genome copies [GC] or 1.9 × 10^7^ GC) were administered intravitreally; then, dose- and time-dependent in vivo transduction was evaluated by detecting mCherry expression through fluorescence fundus imaging and confocal microscopic imaging of retina cross-sections (Figure 5A). Efficient transduction was evident: mCherry expression was first detected at 2 weeks after intravitreal injection, reached a maximum at week 3, and was stable through week 5; greater transduction was achieved at the higher titer (Figure 5B). The corresponding OCT images of the representative fundus images show no substantial difference in anatomical structure of the retina cross-sections at all viral doses tested (Figure 5B).

### 2.4. Localization of Transduced Cells

We next determined whether the AAV8-*Ephx2* shRNA viral vector efficiently and specifically targeted photoreceptor and RPE cells in vivo. At week 5, murine eyes were enucleated for cryosectioning followed by microscopic assessment and immunohistochemistry. Murine eye cryosections showed mCherry expression localized most strongly to the photoreceptor and RPE layers (Figure 5C). At the lower titer, mCherry expression was limited to the ONL at a low level and was absent in the RPE, whereas the higher titer resulted in more mCherry positive cells in both photoreceptor and RPE cell layers. These sections were then stained with antibodies for markers of rods, cones and RPE cells, verifying that these cell types were transduced by intravitreal injection of AAV8 (Figure S3). This transduction pattern was largely consistent with that obtained using an AAV8 GFP vector from another supplier (Figure S4).

### 2.5. Suppression of L-CNV by AAV8-Ephx2 shRNA

**Figure 6:**
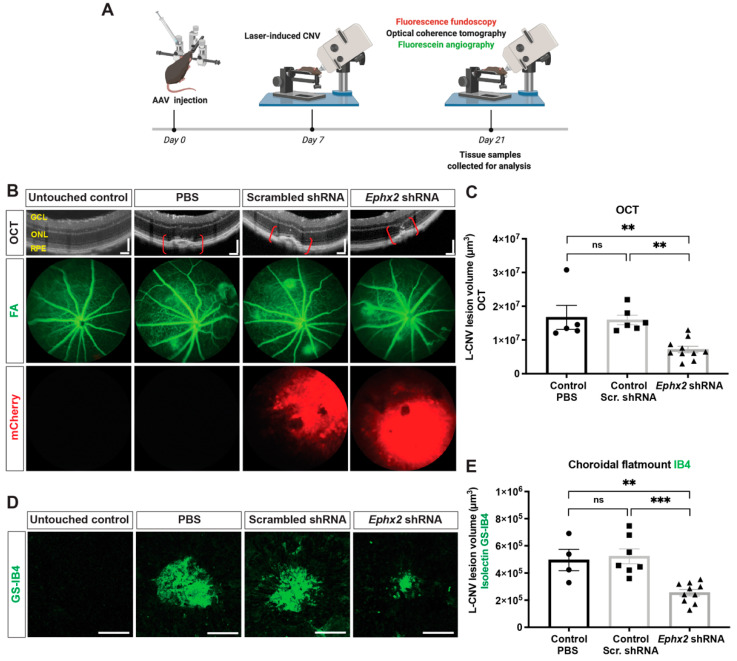
AAV8-delivery of shRNA against sEH suppresses L-CNV. (**A**) Study design and experimental timeline used to assess in vivo efficacy of AAV8-*Ephx2* shRNA. Seven days post intravitreal injection of viral vectors encoding *Ephx2* shRNA or scrambled shRNA or PBS vehicle, mice were treated with laser to induce CNV. Transduction level (mCherry fluorescence) and neovascular volume were assessed by weekly noninvasive ophthalmic imaging tools: fundoscopy and optical coherence tomography (OCT) and fluorescein angiography (FA). On day 21 (14 days post laser), retina and choroid tissue of each treatment group were harvested for choroidal flatmounts. (**B**) Representative OCT, FA (green) and AAV8 (mCherry; red) fluorescence fundus images on day 21. OCT scale bars = 100 µm. (**C**) CNV lesion volume calculated as an ellipsoid from OCT images. (**D**) Representative images from confocal microscopy of *Griffonia simplicifolia* isolectin B4 (GS-IB4; green) stained CNV lesions. Scale bars = 100 µm. (**E**) CNV lesion volume from Z-stack confocal images, showing reduction in CNV lesions with *Ephx2*-shRNA. Mean ± SEM, *n* = 4–10 mice, each data point represents an average of 2–3 L-CNV lesions per eye, one-way ANOVA with Tukey’s post hoc tests (ns, non-significant; ** *p* < 0.01; *** *p* < 0.001). Scr, scrambled. See also Figure S5.

We then evaluated whether intravitreal injection of AAV8-*Ephx2* shRNA would suppress the formation of CNV (Figure 6A). At day 0, mice received intravitreal injection of 1.9 × 10^7^ GC of AAV8-*Ephx2* shRNA, AAV8-scrambled shRNA control, or PBS control (Figure S5). At day 7, laser photocoagulation was performed to induce CNV. At day 21 (day 14 post laser), fluorescence fundoscopy was performed to assess transduction, and to visualize L-CNV and consequent vascular leakage, in vivo optical coherence tomography (OCT) imaging and fluorescein angiography (FA) were performed. Notably, AAV8-*Ephx2* shRNA reduced vascular leakage qualitatively as shown through FA imaging, and CNV lesion volume which was quantitatively assessed based on OCT images (Figure 6B,C). Ex vivo CNV volume measurements at day 21 (14 days post laser) likewise revealed a statistically significant attenuation in CNV in mice injected with AAV8-*Ephx2* shRNA compared to scrambled shRNA and PBS controls (Figure 6D,E).

### 2.6. AAV8-Ephx2 shRNA Inhibition of sEH Expression In Vivo

**Figure 7:**
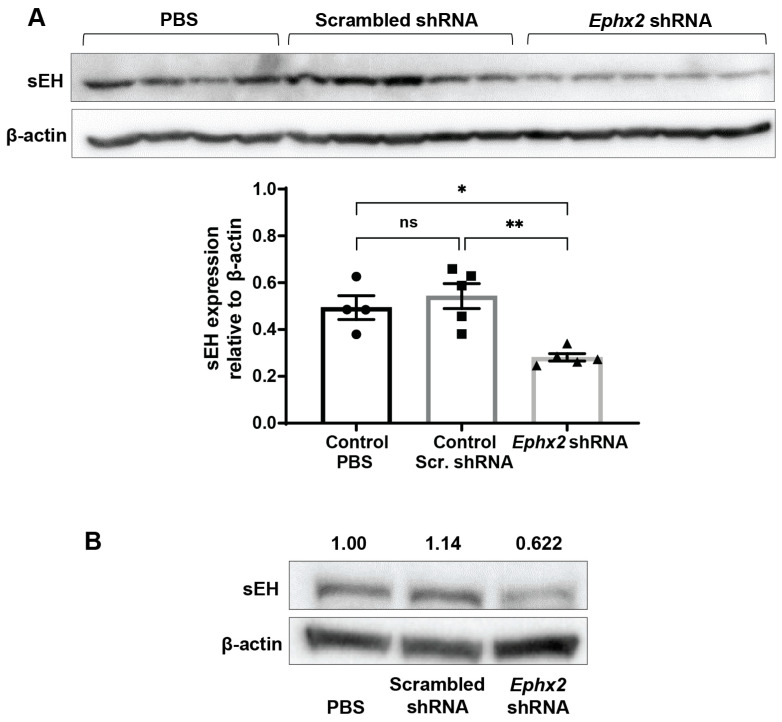
AAV8-*Ephx2* shRNA reduces sEH expression level. Inhibitory effect of AAV8-*Ephx2* shRNA on sEH expression was determined in tissues collected 21 days post intravitreal injection of 1.9 × 10^7^ GC of AAV8-*Ephx2* shRNA, AAV8-scrambled shRNA control or PBS control. (**A**) sEH expression in retina. Each lane represents a single retina sample, with quantification by densitometry. Mean ± SEM, *n* = 4–5 retinas, one-way ANOVA with Tukey’s post hoc tests (ns, non-significant; * *p* < 0.05; ** *p* < 0.001). See Figure S6. (**B**) sEH expression in RPE/choroid. Each lane represents pooled tissue samples from *n* = 3–4 eyes. See Figure S7. Intensity relative to PBS control, normalized to β-actin, is indicated.

To verify that the detected reduction in CNV volume in virally transduced eyes coincided with a reduction in sEH expression, immunoblot analysis was performed using total protein obtained from retina and RPE/choroid isolated from mice injected with AAV8-*Ephx2* shRNA, AAV8-scrambled shRNA control, and PBS control. Compared to the PBS control and scrambled shRNA control, mice administered AAV8-*Ephx2* shRNA had markedly reduced retinal and RPE/choroid sEH expression (Figure 7, Figures S6 and S7). These findings support that AAV8-mediated ocular delivery of shRNA targeting *Ephx2* can lead to depletion of the target gene.

### 2.7. Inhibition of CNV-Related Inflammatory Gene Expression by AAV8-Ephx2 shRNA

**Figure 8:**
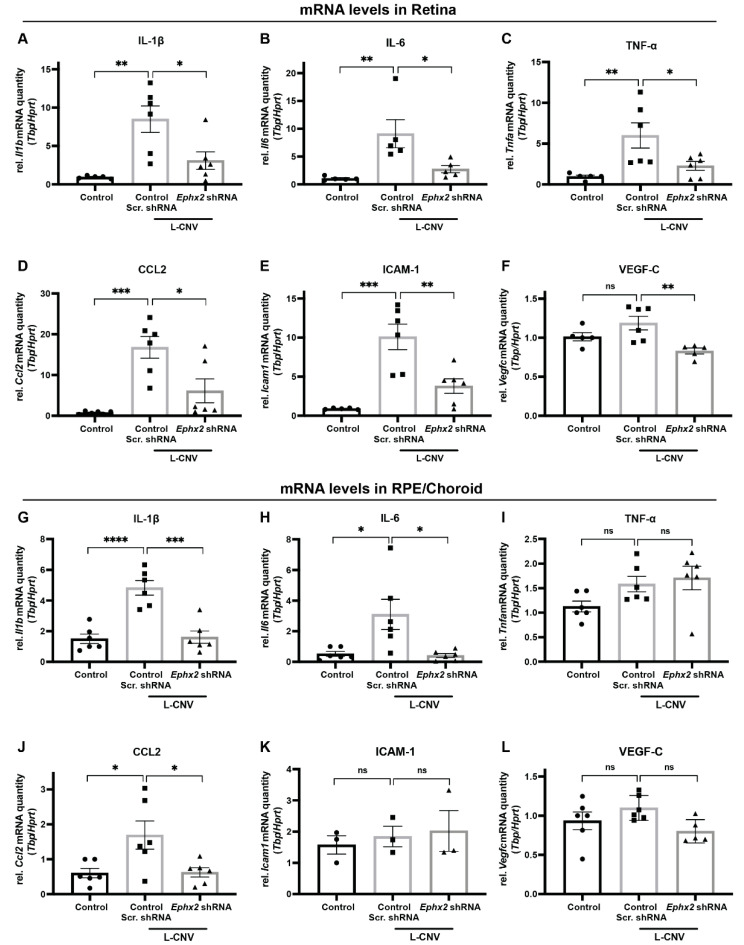
Inhibition of CNV-related inflammatory molecule expression by AAV8 delivery of *Ephx2* shRNA. mRNA expression levels of inflammatory molecules (**A**,**G**) *Il1b*, (**B**,**H**) *Il6*, (**C**,**I**) *Tnfa*, (**D**,**J**) *Ccl2*, (**E**,**K**) *Icam1*, and (**F**,**L**) *Vegfc* in the retina (**A**–**F**) or RPE/choroid (**G**–**L**) of untreated control and L-CNV mice transduced with control scrambled shRNA or *Ephx2* shRNA. qPCR data for the indicated genes relative to *Tbp* and *Hprt* expression, normalized to a single control sample. Mean ± SEM, *n* = 3–6 biological replicates (each data point represents pooled tissue from 2 eyes), one-way ANOVA with Tukey’s post hoc tests (ns, non-significant; * *p* < 0.05; ** *p* < 0.01; *** *p* < 0.001; **** *p* < 0.0001). RPE, retinal pigment epithelium; Scr, scrambled.

To investigate the mechanisms by which *Ephx2* knockdown attenuates CNV formation, we isolated RNA from retina and RPE/choroid tissues of untouched eyes and eyes treated with either AAV8-scrambled shRNA control or AAV8-*Ephx2* shRNA in the L-CNV model. Through the stabilization of anti-inflammatory and pro-resolving EpFAs, inhibition of sEH has shown anti-inflammatory effects in various models of inflammation [20,26,27]. In the context of CNV, sEH-regulated EpFAs suppressed CNV, and modulated leukocyte rolling velocity by changing the expression of adhesion molecules on the surfaces of leukocytes and in the CNV lesions [20]. Given this, the changes in mRNA expression levels of CNV relevant inflammatory molecules were analyzed by qPCR. Compared to scrambled shRNA control, we found that *Ephx2* shRNA-treated retinas had significantly normalized mRNA levels of inflammatory cytokine genes induced by L-CNV, *Il1b*, *Il6*, and *Tnfa* (Figure 8A–C), and cell adhesion molecules, *Ccl2* and *Icam1* (Figure 8D,E). The inhibitory effect was also observed on the mRNA level of *Vegfc* in treated retinas (Figure 8F). In treated RPE/Choroid, *Ephx2* shRNA likewise significantly reduced mRNA levels of *Il1b*, *Il6* and *Ccl2* (Figure 8G,H,J), whereas no significant differences were detected in *Tnfa*, *Icam1* and *Vegfc* (Figure 8I,K,L), which were also not significantly upregulated by L-CNV in the RPE/choroid.

### 2.8. sEH Lipid Substrate 19,20-EDP Is Antiangiogenic in the Eye

**Figure 9:**
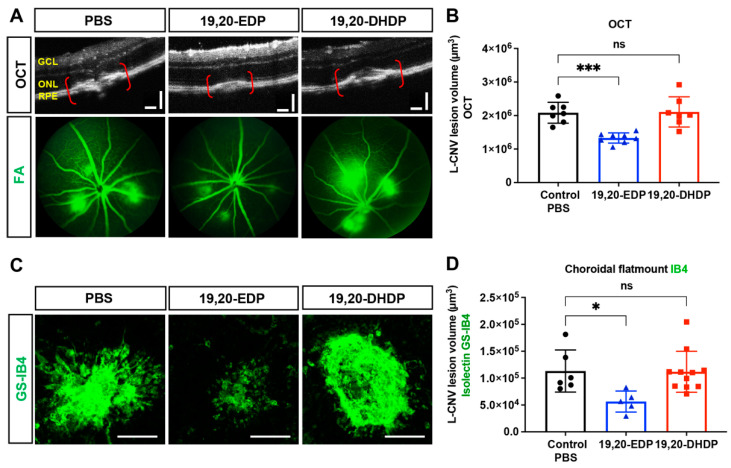
Lipid substrate of sEH, 19,20-EDP, is antiangiogenic when delivered intravitreally. A single intravitreal injection of 19,20-EDP at a final concentration of 100 µM at the time of laser treatment reduces L-CNV lesion volume compared to vehicle (PBS + 0.5% DMSO). Intravitreal 19,20-DHDP (100 µM) did not significantly change lesion volume. (**A**) Representative OCT and FA (green) fundus images on day 14 after laser. Scale bars = 100 µm. (**B**) CNV lesion volume calculated as an ellipsoid from OCT images. (**C**) Representative images from confocal microscopy of *Griffonia simplicifolia* isolectin B4 (GS-IB4; green) stained CNV lesions. Scale bars = 100 µm. (**D**) CNV lesion volume from Z-stack confocal images. Mean ± SEM, *n* = 5–11. Each data point represents an average of 2–3 L-CNV lesions per eye, one-way ANOVA with Dunnett’s post hoc test (ns, non-significant; * *p* < 0.05; *** *p* < 0.001).

To assess the relevance of local lipid substrates of sEH to neovascularization, we tested the effects of intraocular administration of 19,20-EDP and 19,20-DHDP on L-CNV (Figure 9). Mice received intravitreal injection of the ω-3 derived sEH substrate and product, 19,20-EDP and 19,20-DHDP, respectively, and underwent L-CNV. We observed that 19,20-EDP had an inhibitory effect on L-CNV assessed 14 days after injection, but no substantial effect was seen with 19,20-DHDP, suggesting that sEH can mediate L-CNV through hydrolysis of 19,20-EDP rather than production of 19,20 DHDP.

## 3. Discussion

Previously, we identified sEH as a therapeutic target for CNV [9]. The present study demonstrates (1) expression and localization of *EPHX2*/*Ephx2* mRNA in human and murine CNV using RNAscope in situ hybridization, and (2) efficacy of AAV8-*Ephx2* shRNA in suppression of CNV in a murine L-CNV model.

Although we previously demonstrated that sEH is upregulated in murine L-CNV in the outer retina (including rod photoreceptors) compared to controls [9], others showed that sEH was primarily localized to Müller glia and astrocytes in the developing retina and in the oxygen-induced retinopathy model of retinal neovascularization [21]. Co-immunostaining of sEH with cell type markers in the current study confirmed that sEH is overexpressed in photoreceptors and RPE cells in areas with degenerative changes in human nAMD specimens. RNAscope ISH allowed further validation of these findings. *EPHX2/Ephx2* mRNA was detected throughout the retina, concentrated within the ONL of photoreceptors and in RPE. In nAMD retinas compared to controls, *EPHX2* mRNA was highly expressed, with notable signals in the apical RPE. Single-cell RNAseq data support this finding: *EPHX2* mRNA is more enriched in rod and RPE cells than other retinal cell types in human retinas obtained from a mixed pool of donors (dataset: all_retina_rpe_chor; Singlecell-eye.org). In murine retinas, we observed *Ephx2* mRNA mostly within the INL, photoreceptors, and moderately in RPE. Expression increased in L-CNV retinas most notably in photoreceptors and RPE, which mirrors observations in human nAMD retinas, although *EPHX2* signals were more prominent in human retinas than in mice.

The localization of *Ephx2* in Müller glia was consistent with other studies that previously reported sEH protein expression in these cells [21,22], although we saw little Müller cell co-staining at the protein level in L-CNV in our previous study [9]. Müller glia closely interact with the inner retinal vasculature [28] and therefore play an outsized role upon inner retina injury in retinopathies [28] such as diabetic retinopathy and retinopathy of prematurity, in models of which cellular expression of sEH has been previously profiled [21,29]. Conversely, the RPE is intimately associated with choroidal vasculature in the outer retina, and the RPE and choroid provide a supporting system for photoreceptors that are implicated in CNV [30]. Altogether, overexpression of sEH at the protein and mRNA level in CNV and disease-relevant cell types indicates a role of sEH in AMD pathophysiology and provides a rationale to target these cell types for developing therapies.

We previously demonstrated that pharmacological inhibition of sEH using small molecules delivered intravitreally suppresses L-CNV [9]. Oral administration of an sEH inhibitor was also reported to reduce CNV lesions [20]. Here, we pursued a genetic approach to evaluate whether depletion of sEH can phenocopy the anti-angiogenic effects observed with small molecule inhibitors [31]. Delayed retinal angiogenesis was seen in systemic sEH knockout mice and Müller cell specific sEH knockout mice [21]. However, intraocular sEH knockdown and its effect on CNV have not been studied before, despite being critical in sEH target validation, since sEH inhibition can have varying effects on angiogenesis depending on tissue levels of PUFAs that are parent to EpFAs.

Intravitreally administered AAV8-*Ephx2* shRNA efficiently transduced photoreceptors and RPE, at least at the doses and timepoints assessed; long-term efficacy and whether sEH reduction has a “field effect” or is only useful if transduced in areas of active CNV remain to be explored. This outer-retinal transduction pattern has been seen before for intravitreal delivery of AAV8 vectors [32], although intravitreal delivery more often results in higher GCL transduction. Importantly, we confirmed a similar transduction pattern with a reporter AAV from another supplier, delivered by another experimenter in a separate experiment (Figure S4). AAV8-*Ephx2* shRNA substantially reduced retinal and RPE/choroid sEH protein levels and suppressed the progression of CNV.

AAV8-*Ephx2* shRNA had inhibitory effects on inflammatory target molecules that were upregulated by L-CNV. The differential downregulation of target molecules between the treated retinas and RPE/choroid could be due to involvement of different signaling pathways in the microenvironment that are also dependent on CNV progression over time. Cell adhesion molecules are expressed differently between retina and choroid tissue at different timepoints of murine L-CNV progression [33]. Additionally, the presence of untransduced cells and choroid/sclera tissue in the RPE/choroid samples could have led to underestimation of the vector’s effects, therefore resulting in differential downregulation of gene expression between the retina and RPE/choroid.

Although the pathogenesis of nAMD is not fully understood, it is thought that the initial stage of CNV is characterized by changes in the microenvironment due to RPE and photoreceptor cells producing growth factors that incite angiogenesis [34,35]. In addition, inflammatory processes such as microglia activation, leukocyte invasion, oxidative stress and lipid deposition are all implicated in RPE dysfunction in AMD [36], leading to photoreceptor/RPE degeneration [37,38]. The resulting production of inflammatory mediators can promote endothelium activation, resulting in increased adhesion molecules and vascular permeability, and further promoting production of angiogenic factors. IL-1β has potent chemotactic and angiogenic properties in addition to initiating acute inflammatory responses [39], and its implication in nAMD pathogenesis is established [40,41]. In AMD, the choroidal vasculature and the RPE secrete IL-1β [41]. IL-1β also activates and enhances production of IL-6 and CCL2 [42,43]. Importantly, IL-6 has potent proangiogenic properties and is involved in L-CNV development, along with numerous correlative observations in human nAMD samples [44,45,46]. Likewise, TNF-α is implicated in human nAMD and murine L-CNV [47]. CCL2 is a member of the chemokine family that directs leukocyte migration [48]. Its expression is very low in healthy retina and RPE [49], but significantly upregulated with acute inflammation, age, and oxidative stress in the RPE [50,51]. Considering the role of CCL2 in leukocyte migration, the inhibitory effect on *Ccl2* expression by AAV8-*Ephx2* shRNA is intriguing and could corroborate other studies that reported that sEH inhibition and the ω-3 EpFAs (lipid substrates of sEH) interfered with leukocyte invasion into CNV lesions [20].

Without longitudinal collection of tissue and analysis, a limitation of the present study is that our data are only representative of changes in the chorioretinal microenvironment at day 14 post L-CNV. This may not fully capture the effects of AAV8-*Ephx2* shRNA on the molecular mechanisms implicated in temporal progression of CNV. Moreover, the expression and therapeutic relevance of sEH in the aging mouse eye and long term after L-CNV remain to be assessed. In addition, the present study does not identify changes in sEH-dependent lipid metabolites. However, our previous lipid-profiling study demonstrated that the ratio of 19,20-EDP to 19,20-DHDP was significantly decreased in L-CNV chorioretina, indicative of increased sEH activity, and the pharmacological inhibition of sEH normalized this ratio, providing evidence that sEH inhibition potentiates 19,20-EDP in the eye [9]. Moreover, we show here that intraocular delivery of 19,20-EDP is antiangiogenic in the eye, further implicating an antiangiogenic mechanism of substrate stabilization when sEH activity is reduced. This finding corroborates the antiangiogenic effects of dietary intake of ω-3 PUFAs and 19,20-EDP in animal models of ocular angiogenesis [20,52]. DHA-derived EDP is the major target of sEH in the eye, as DHA is the most abundant PUFA in the retina [53], constituting 50–60% of the total fatty acids in the photoreceptors, in contrast to most tissues that contain only a small portion (∼5%) of their fatty acids as DHA [54,55,56].

The present study builds support for sEH as an important target involved in inflammatory and angiogenic processes in CNV. The study also highlights exciting potential in targeting sEH with an AAV-mediated gene therapeutic approach that presents stable depletion of the therapeutic target in the disease relevant cell types, which may address shortcomings of pharmacological interventions [57]. Given the involvement of both angiogenesis and inflammation in the pathogenesis of CNV, stabilization of pro-resolving epoxy fatty acids through sEH inhibition could be a promising therapeutic strategy for CNV underlying nAMD.

## 4. Materials and Methods

### 4.1. Animals

All mouse experiments were approved by the Institutional Animal Care and Use Committee, Indiana University School of Medicine (protocol code 19059, approved 10 July 2019) and followed the guidelines of the Association for Research in Vision and Ophthalmology (ARVO) Statement for the Use of Animals in Ophthalmic and Visual Research. Wild-type C57BL/6J male mice, 7 weeks of age were purchased from Jackson Laboratory (Bar Harbor, ME, USA). The mice were housed under standard conditions in the Indiana University Laboratory Animal Resource Center (LARC) [58]. Specifically, the light-dark cycle was 12 h, temperature was 21 ± 1 °C, and humidity was 30–70%. Animals were fed Teklad 2018SX rodent chow and chlorinated water (via Hydropac) ad libitum. Sample sizes for experiments were based on power analyses, and treatments were randomly assigned by cage and animals. The experimenter was masked to treatment during analyses.

### 4.2. Human Samples

Formalin fixed paraffin-embedded (FFPE) retinal sections were prepared from human donor eyes from nAMD and control subjects with no documented ocular pathology that were obtained from the National Disease Research Interchange (NDRI; Philadelphia, PA, USA). All human sample tissues were anonymized with full ethical approval for research use. Normal control subjects included an 81 y.o. female, a 68 y.o. male and a 79 y.o. female. nAMD subjects included a 72 y.o. female, a 79 y.o. male, and an 81 y.o. male.

### 4.3. Immunofluorescence

For immunostaining of FFPE human eye sections, sections were deparaffinized, rehydrated and underwent heat-induced antigen retrieval in which tissue slides were immersed in citrate buffer at pH 6.0 (Thermo Scientific, Waltham, MA, USA) and heated (>95 °C) for 15 min. Sections were then washed with TBS and blocked in 10% DAKO diluent in 1% BSA in TBS-0.1% Tween-20 for an hour at room temperature, then incubated overnight at 4 °C with primary antibodies: mouse anti-sEH, A5 (1:100; Santa Cruz, Dallas, TX, USA), PNA biotin conjugate, L6135 (1:250; Sigma, St. Louis, MO, USA), WGA Alexa Fluor 647 conjugate, W32466 (1:250; Thermo Fisher, Waltham, MA, USA) and rabbit anti-RPE65, ab231782 (1:400; Abcam, Cambridge, UK). The primary antibody and lectin details are summarized in Table S1. The specificity of PNA binding to cone matrix domains and that of WGA binding to rod matrix domains in human retinas have been well characterized in the literature [59,60]. Sections were then incubated with the following secondary antibodies for an hour at room temperature: goat anti-mouse IgG (H+L) Alexa Fluor 555, Streptavidin DyLight 488, or goat anti-rabbit IgG (H+L) Alexa Fluor 480 (each at 1:400, Thermo Fisher). After a brief wash in TBS and dehydration in sequential ethanol and xylene baths, sections were mounted with Vectashield mounting medium plus DAPI (Vector Laboratories, Newark, CA, USA).

For the immunostaining of AAV-transduced fixed-frozen mouse retina, cryosections were prepared as described below under the description of embedding and cryosectioning. Sections were brought to room temperature and washed with PBS, then blocked in 2.5% BSA, 0.3% Triton-X 100 in PBS for 1 h at room temperature. The sections were incubated with the following primary antibodies overnight at 4 °C: mouse anti-rhodopsin, ab3424 (1:300; Abcam) and rabbit anti-cone arrestin, ab15282 (1:500; Millipore, Burlington, MA, USA). Subsequently, sections were incubated with the following secondary antibodies for 1 h at room temperature: goat anti-mouse IgG (H+L) Alexa Fluor 555 or goat anti-rabbit IgG (H+L) Alexa Fluor 480 (each at 1:400, Thermo Fisher). Sections were washed in PBS and mounted with Vectashield mounting medium plus DAPI. Images were acquired using the 20× objective of an LSM 700 laser scanning confocal microscope with ZEN imaging software (Zeiss, Thornwood, NY, USA).

### 4.4. Mean Fluorescence Intensity Analysis

Mean fluorescence intensity (MFI) of sEH in human retinal sections was quantified as described [61]. Briefly, laser settings for gain, intensity, pinhole, and digital offset for all channels remained consistent during image acquisition on the LSM700 confocal microscope. Using ImageJ software, a freehand selection was used to select the outer retina (outer plexiform layer through RPE) or whole retina (GCL through RPE) as a region of interest (ROI). The mean intensity divided by the number of pixels of the ROI was calculated. The MFI was calculated as: Final MFI = MFI of ROI – MFI of background, in which the background was the area outside of the ROI in the same slide. This analysis was performed by a masked investigator to minimize investigator bias.

### 4.5. RNAscope In Situ Hybridization

RNAscope ISH was performed on FFPE human retina sections and frozen mouse retina sections as directed by the manufacturer (ACD, Advanced Cell Diagnostics, Newark, CA, USA). Briefly, sections were treated with hydrogen peroxide, then underwent antigen retrieval and protease digestion. After the pretreatment, RNAscope Multiplex Fluorescent Assay was performed to hybridize target specific probes. RNAscope Hs-*EPHX2* (Cat No. 558,681) was used to detect human *EPHX2* mRNA. RNAscope Mm-*Ephx2* (Cat No. 558,701) and Mm-*Apoe* (Cat No. 313,271) were used to detect mouse *Ephx2* mRNA and *Apoe* mRNA, respectively, along with ACD universal negative control probe targeting the *DapB* gene (accession # EF191,515) from the *Bacillus subtilis* strain SMY. After the hybridization, a three-step amplification process was performed followed by development of channel specific signal and binding of 570 Opal fluorophore, SKU FP1488001KT (1:500, Akoya Biosciences, Marlborough, MA, USA) for *EPHX2*/*Ephx2* mRNA and 520 Opal fluorophore, SKU FP1487001KT (1:500, Akoya Biosciences) for *Apoe* mRNA. Sections were then counterstained with DAPI for nuclear identification and coverslipped using antifade mountant, Fluoromount-G (SouthernBiotech, Birmingham, AL, USA). Slides were imaged on an LSM 700 laser scanning confocal microscope with ZEN imaging software (Zeiss). To allow for quantitative and qualitative comparisons, standardized settings remained constant. Using a 20× objective, images providing coverage across retina, RPE and choroid were obtained. In addition, a higher magnification oil immersion objective was used to acquire cell layer specific detection of mRNA signals.

### 4.6. Generation of AAV8 Vectors

Mouse *Ephx2* shRNA (5′-GCCATGGAATTGCTGTGTAAGTTCAAGAGACTTACACAGTAA TTCCATGGCTTTTTT-3′) and non-targeting scrambled shRNA constructs were cloned into a pAV vector under the control of a U6 promoter, and these were packaged into AAV serotype 8 by Vigene Biosciences (Rockville, MD, USA). The pAV vector also contains a CMV-mCherry reporter. Briefly, the transfer plasmid/s, the Rep/Cap and the Helper plasmid were co-transfected into HEK293 cells. After 70 h, the cells were collected and lysed by a freeze and thaw cycle. The crude lysate was clarified by centrifugation, and the viral particles were purified via ultracentrifugation in an iodixanol gradient to remove impurities and empty viral capsids for safe use in vivo. After collection from the gradient, the viral titer was then determined using quantitative PCR with primers to the inverted terminal repeats present in the viral genome, and viral particles were buffer exchanged into 1 × PBS, 0.01% Pluronic F-68 and stored at −80 °C prior to use. A control ready-made AAV8-CMV-eGFP virus (Cat. No. 7061) was also purchased from Vector Biolabs (Malvern, PA, USA).

### 4.7. Intravitreal Injection

Pupils of both eyes were dilated with topical drops of 1% tropicamide (Henry Schein Inc., Melville, NY, USA) and 2.5% phenylephrine ophthalmic solution (Akorn, Lake Forest, IL, USA). Mice were anesthetized by intraperitoneal injections of 90 mg/kg ketamine hydrochloride and 5 mg/kg xylazine mixture. Tetracaine hydrochloride 0.5% ophthalmic solution (Bausch & Lomb, Laval, QC, Canada) was applied for topical anesthesia. Anesthetized mice were positioned on a stereotaxic instrument with a head holder (World Precision Instruments, Sarasota, FL, USA), and mice were held in position by inserting the ear and tooth bars to stabilize the head of the anesthetized mouse. Mice were then placed on the platform of a stereo microscope (SMZ745, Nikon, Melville, NY, USA). A small incision was made through the limbus using a 30 G insulin syringe needle. A Hamilton syringe with 33 G needle was inserted carefully into the vitreous body without damaging the lens. Then, 4.9 × 10^6^ GC or 1.9 × 10^7^ GC of viral vectors or PBS vehicle control in a total volume of 0.5 µL was intravitreally injected. Upon determining successful transduction efficiency at the higher viral dose, three groups of 25 mice were injected with PBS vehicle, 1.9 × 10^7^ GC of AAV8-scrambled shRNA control, or AAV8-*Ephx2* shRNA in their right eyes. In total, 22 of the 75 treated mice were used for the L-CNV assessment, 14 mice were used for the immunoblot analysis, and 30 mice were used for the gene expression analysis. Overall, 9 mice were excluded due to injection failure noted by volume reflux, bleeding, or cataract development.

### 4.8. Embedding and Cryosectioning

Corneas of enucleated mouse eyes were punctured with a 29 G needle, and the whole eye was fixed in 4% formaldehyde in PBS at 4 °C overnight. Fixed whole eye was dissected to remove cornea and isolate posterior cup of chorioretinal tissue, which was rinsed in PBS and immersed in a sucrose gradient. Tissue was then transferred to a cryomold containing Optimal Cutting Temperature compound (Fisher Scientific, Hampton, NH, USA) and stored at −80 °C until sectioning. Samples were sectioned using a cryostat (Leica Biosystems, Wetzlar, Germany) at −20 °C, with section thickness of 10 µm, and mounted on SuperFrost Plus Slides (Fisher Scientific).

### 4.9. Laser-Induced Choroidal Neovascularization

At day 7 post intravitreal injection of AAV, pupils were dilated, and mice anesthetized as above. Corneas were kept lubricated with 2.5% Gonak hypromellose ophthalmic solution (Akorn). The anesthetized mouse was placed on the stage of a Micron IV Laser Injector (Phoenix-Micron, Bend, OR, USA). By adjusting the stage, the mouse was aligned to the camera, and the iris and pupil were brought into focus. The Micron instrument was advanced forward while focusing on retinal features. Eyes were then subjected to 50 μm spot size, 70 ms, 250 mW pulses of the argon green ophthalmic laser coupled to the Micron IV ocular imager, resulting in 3 laser burns per eye approximately two-disc diameters from the optic disc. For experiments in Figure 9, 50 μm spot size, 50 ms duration, and 250 mW pulses of an ophthalmic argon green laser, wavelength 532 nm (Oculight, Iridex, Mountainview, CA, USA), coupled to a slit lamp (Zeiss) were used. Lipids were injected a single time immediately post laser treatment (all in eyes without AAV), delivered intravitreally using a 33 G needle according to the procedure above, in a 0.5 μL volume. Lipids 19,20-EDP or 19,20-DHDP (Cayman Chemical, Ann Arbor, MI) were dissolved in DMSO and diluted in PBS to a final concentration of 0.5% (*v*/*v*) DMSO. Vehicle alone (PBS + 0.5% (*v*/*v*) DMSO) was used as a control.

For all L-CNV studies, gaseous bubble formation at each laser spot, which is indicative of Bruch’s membrane rupture, was required for the lesion to be included in further analyses. We applied consistent exclusion criteria as follows: (1) cataract or any other inherent ocular abnormality, (2) laser-induced choroidal hemorrhage greater than grade 1 [62], and (3) damage of the CNV lesions during tissue dissection.

### 4.10. In Vivo Retinal Imaging

Mice were anesthetized, pupils were dilated, and corneas were lubricated under the same conditions as for L-CNV described above. A Micron III or IV ocular imager was used for in vivo retinal imaging—bright-field fundus, OCT, fluorescent fundus, and fluorescein angiography. Simultaneous bright-field live fundus images and OCT scans were acquired to assess retinal features and to visualize CNV lesion development in cross-sectional retinal layers. Several horizontal and vertical OCT scans per lesion were taken to calculate CNV lesion volume as previously described [63]. Fluorescent fundus imaging of mCherry was performed using a 562/40 nm excitation filter, and GFP using a 469/35 excitation filter and long-pass emission filters, to assess in vivo AAV transduction. Fluorescein angiography by i.p. injection of 5 mL/kg of 10% fluorescein sodium (Fisher Scientific) and imaging using the GFP filter set after 3–5 min was performed to assess vascular leakage resulting from L-CNV. Images were acquired using the manufacturer’s image acquisition software.

### 4.11. Choroidal Flatmounts and Ex Vivo CNV Lesion Quantification

On day 14 post L-CNV induction, mice were euthanized by isoflurane overdose followed by cervical dislocation. The eyes were enucleated and fixed in 4% PFA in PBS for 1 h at 4 °C. The anterior portion including lens and the retina were removed, then the posterior eyecups were dissected out and underwent further fixation in 4% PFA in PBS overnight. The fixed eye cups were washed in blocking buffer (0.3% Triton X-100, 5% bovine serum albumin (BSA) in PBS) for 2 h at 4 °C. The eyecups were then stained for vasculature using Alexa Fluor 488 conjugated *Griffonia simplicifolia* isolectin B4 (GS-IB4; Molecular Probes, Thermo Fisher Scientific) at 1:250 dilution in buffer containing 0.3% Triton X-100, 0.5% BSA in PBS, overnight at 4 °C. The posterior eyecups were washed three times with PBS and mounted in Vectashield fluorescent mounting medium (Vector Laboratories) and coverslipped. Confocal imaging and analysis of L-CNV lesion volume were performed as previously described [64,65]. Choroidal flatmount images were acquired using a Zeiss LSM 700 laser scanning confocal microscope with ZEN imaging software. The area measurements and volume calculations were performed using the freehand tool in ImageJ software.

### 4.12. Immunoblot Assay

Mice were sacrificed 21 days post injection of 1.9 × 10^7^ GC AAV8-*Ephx2* shRNA or AAV8-scrambled shRNA control, or PBS vehicle control. The eyes were enucleated, placed immediately in ice-cold 1× PBS, and dissected under a microscope to remove optic nerve and periocular tissue, followed by removal of cornea and lens to obtain the posterior cup of the chorioretina. The neuroretina and RPE/choroid tissues were gently separated and then snap frozen. Tissue lysates were prepared by homogenizing the tissue in RIPA buffer (Sigma-Aldrich) with protease inhibitor cocktail (Roche, Basel, Switzerland), and then centrifuged at 12,000× *g* for 15 min at 4 °C. The supernatant was recovered, and the total protein concentration was determined using the Protein Assay Dye Reagent Concentrate (Bio-Rad, Hercules, CA, USA). Immunoblotting was performed as previously described [9]. Electrotransferred PVDF membranes were incubated with murine antibody against sEH (A-5, sc-166561, Santa Cruz, 1:1000) overnight at 4 °C. As a loading control, membranes were incubated with murine antibody against β-actin (AC-40, Sigma-Aldrich, 1:5000) for 1 h at room temperature. Prior to immunoblotting for β-actin, Restore Western Blot Stripping Buffer (Thermo Scientific) was used according to the manufacturer’s protocol to strip the membrane. Secondary antibody was horseradish peroxidase (HRP)-conjugated goat-anti-mouse IgG (1:10,000, Jackson ImmunoResearch, West Grove, PA, USA) for 1 h at room temperature. Signals were detected using ECL immunoblotting detection reagents (Amersham, Amersham, UK) on a c600 imaging system (Azure Biosystems, Dublin, CA, USA). The bands were quantified by densitometry using ImageJ software.

### 4.13. Mouse Eye RNA Extraction and qPCR

Mice treated as above were sacrificed 21 days post AAV injection. The eyes were enucleated, and the retina and choroid tissues were separated using the dissection procedure described above. The retina or choroid tissue was placed in 500 µL of TRIzol reagent and homogenized by a Kimble Pellet Pestle tissue grinder and vortexing. Total RNA was isolated using the TRIzoL reagent according to the manufacturer’s instructions (Thermo Scientific). To clean up RNA, RNA Clean & Concentrator kit was used according to the manufacturer’s protocol (Zymo Research, Irvine, CA, USA). cDNA synthesis was carried out on 400 ng total RNA using the iScript cDNA synthesis kit (Bio-Rad) as per the manufacturer’s protocol. qRT-PCR reactions were prepared using TaqMan Fast Advanced Master Mix, TaqMan gene specific probe (Thermo Fisher) and cDNA in nuclease free water. All TaqMan probe sets are listed in Table S2. A ViiA 7 Real-Time PCR system (Thermo Fisher) was used to perform qPCR under the following conditions: hold at 50 °C for 2 min, hold at 95 °C for 2 min, and 40 cycles of denature at 95 °C for 1 sec and anneal/extend at 60 °C for 20 sec. Quantification was performed by the ΔΔC_t_ method, normalizing to *Hprt* and *Tbp* and comparing to a single control sample.

### 4.14. Statistical Analysis

Data are presented as mean ± SEM; *n* is listed and defined in figure legends. One-way ANOVA with Tukey’s post hoc tests was used to compare treatment groups in the AAV studies, while one-way ANOVA with Dunnett’s post hoc tests was used to compare two treatment groups to vehicle control in the intraocular lipid delivery study. Values of *p* < 0.05 were considered statistically significant. All statistical analyses were performed using Prism 9 software (GraphPad, San Diego, CA, USA) and graphics generated with GraphPad and biorender.com.

## Data Availability

The data presented in this study are available in the article and Supplementary Material. Raw data are available on request from the corresponding author.

## Supplementary Materials

The supporting information can be downloaded at: https://www.mdpi.com/article/10.3390/ijms232415595/s1.
