# Supplementary material for: Decreased Expression of Soluble Epoxide Hydrolase Suppresses Murine Choroidal Neovascularization
Source: Int J Mol Sci. 2022 Dec 9;23(24):15595. doi: 10.3390/ijms232415595 (PMC9779010; doi:10.3390/ijms232415595)
Supplement: Supplementary file 1 [file ijms-23-15595-s001.zip › ijms-2002018-supplementary.pdf]

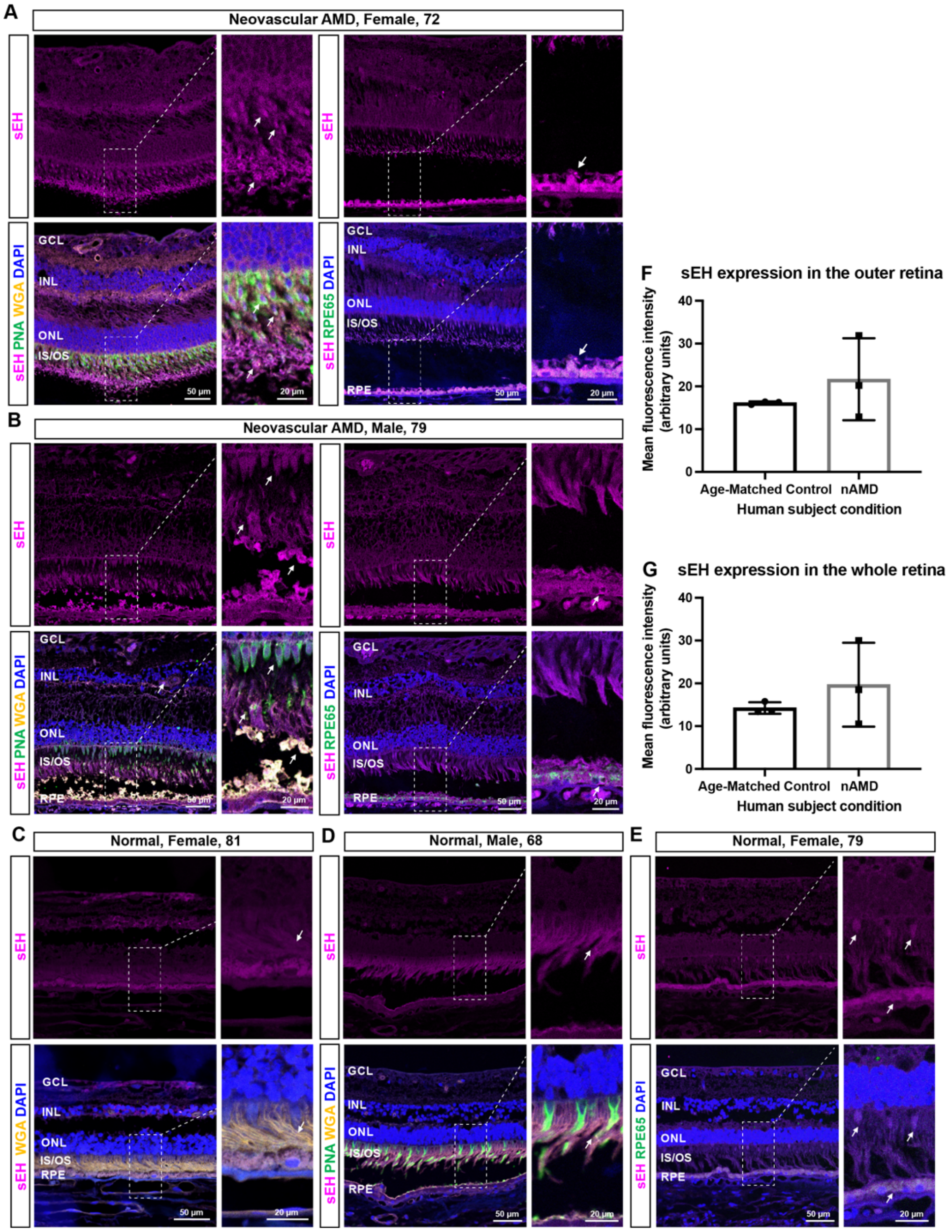

Figure S1. Further images of sEH immunostaining in human retina. Images of retinal sections from two additional human neovascular AMD eyes, (**A**) 72 y.o., female and (**B**) 79 y.o., male; and control eyes (**C**) 81 y.o., female, (**D**) 68 y.o., male, (**E**) 79 y.o., female, stained with DAPI (blue), sEH (magenta), peanut agglutinin (PNA) (cone photoreceptors; green), wheat germ agglutinin (WGA) (rod photoreceptors; yellow), or RPE65 (retinal pigment epithelium; green). Labeling of retinal tissues with PNA and WGA for rods and cones, and with RPE65 show colocalization of sEH in photoreceptors and RPE. In neovascular AMD, sEH is increased in the (**F**) outer and (**G**) total retina as demonstrated by mean fluorescence intensity (MFI) quantification. Insets show higher magnification images of the indicated areas. Scale bars as indicated. GCL, ganglion cell layer; INL, inner nuclear layer; ONL, outer nuclear layer; IS/OS, photoreceptor inner segments/outer segments; RPE, retinal pigment epithelium.

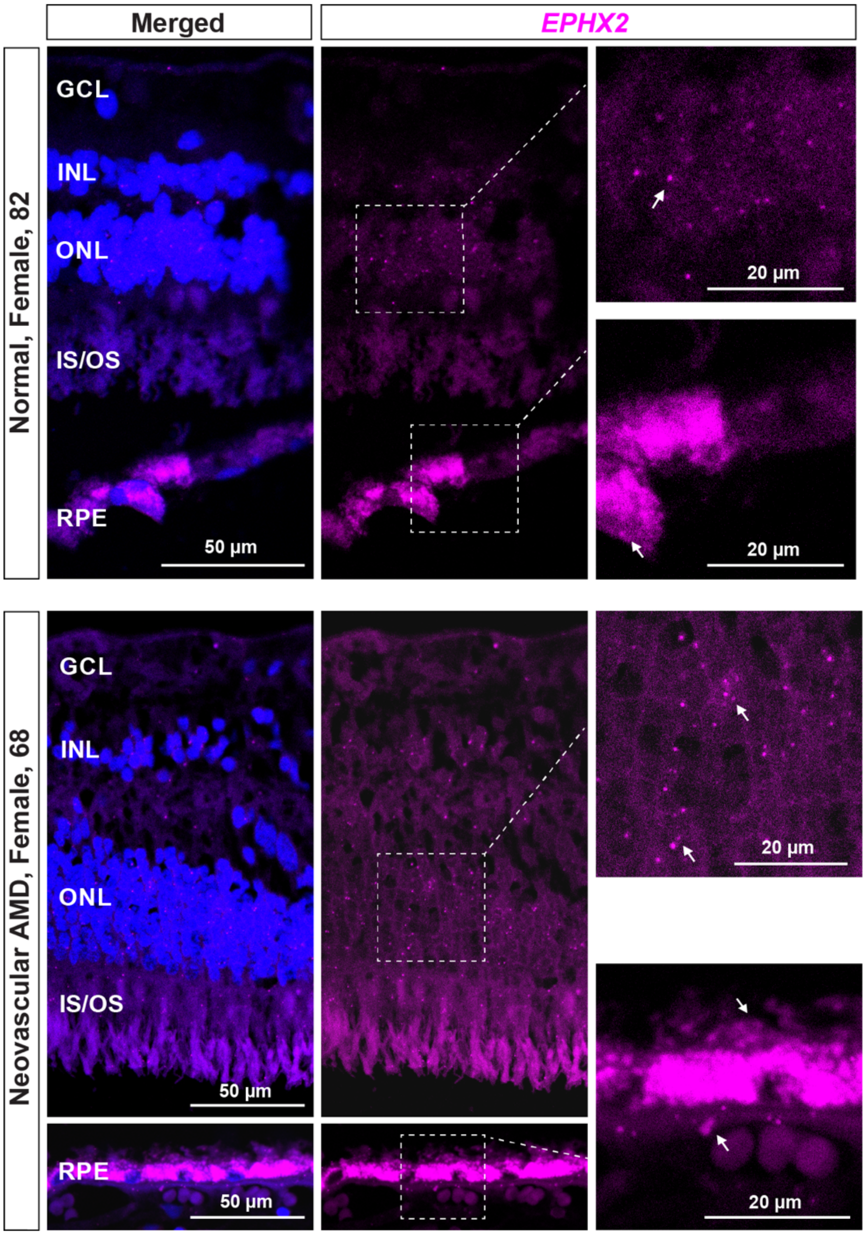

Figure S2. Further images of RNAscope detection of *EPHX2* mRNA in human retina. *EPHX2* encoding sEH is in magenta and nuclei (DAPI) are in blue. Insets show higher magnification images of the indicated areas. Scale bars as indicated. *EPHX*2 mRNA is highly expressed in the photoreceptor cell bodies in the outer nuclear layer and in the RPE. GCL, ganglion cell layer; INL, inner nuclear layer; ONL, outer nuclear layer; RPE, retinal pigment epithelium.

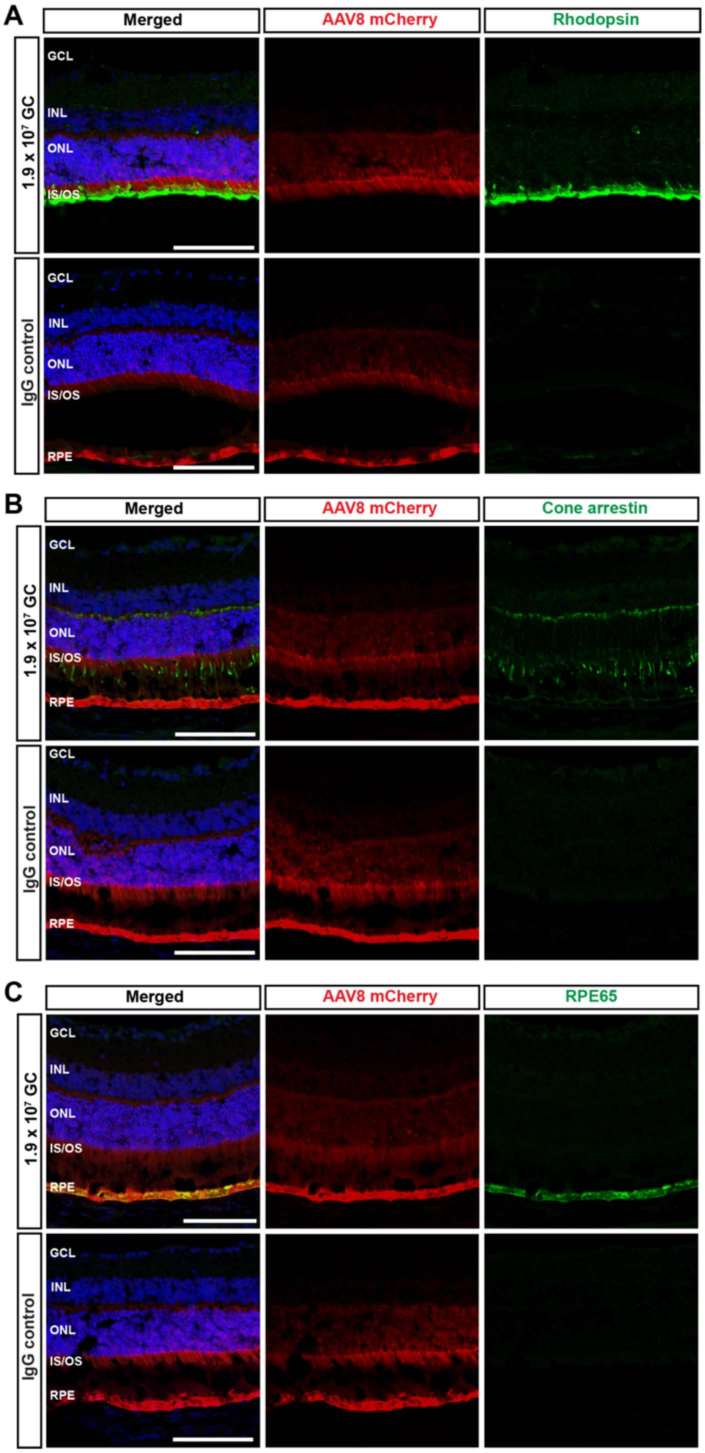

Figure S3. Immunolocalization of AAV8 mCherry with cone arrestin, rhodopsin and RPE65 in AAV8 injected murine eyes. Five weeks after intravitreal injection, AAV8 transduction (mCherry; red) was observed associated with (**A**) rod photoreceptor cells (rhodopsin, which stains outer segments; green), (**B**) cone photoreceptor cells (cone arrestin; green) and (**C**) RPE cells (RPE65; green). Nuclei (DAPI) are blue. GCL, ganglion cell layer; INL, inner nuclear layer; ONL, outer nuclear layer; IS/OS, photoreceptor inner segments/outer segments; RPE, retinal pigment epithelium. Scale bars = 100 μm.

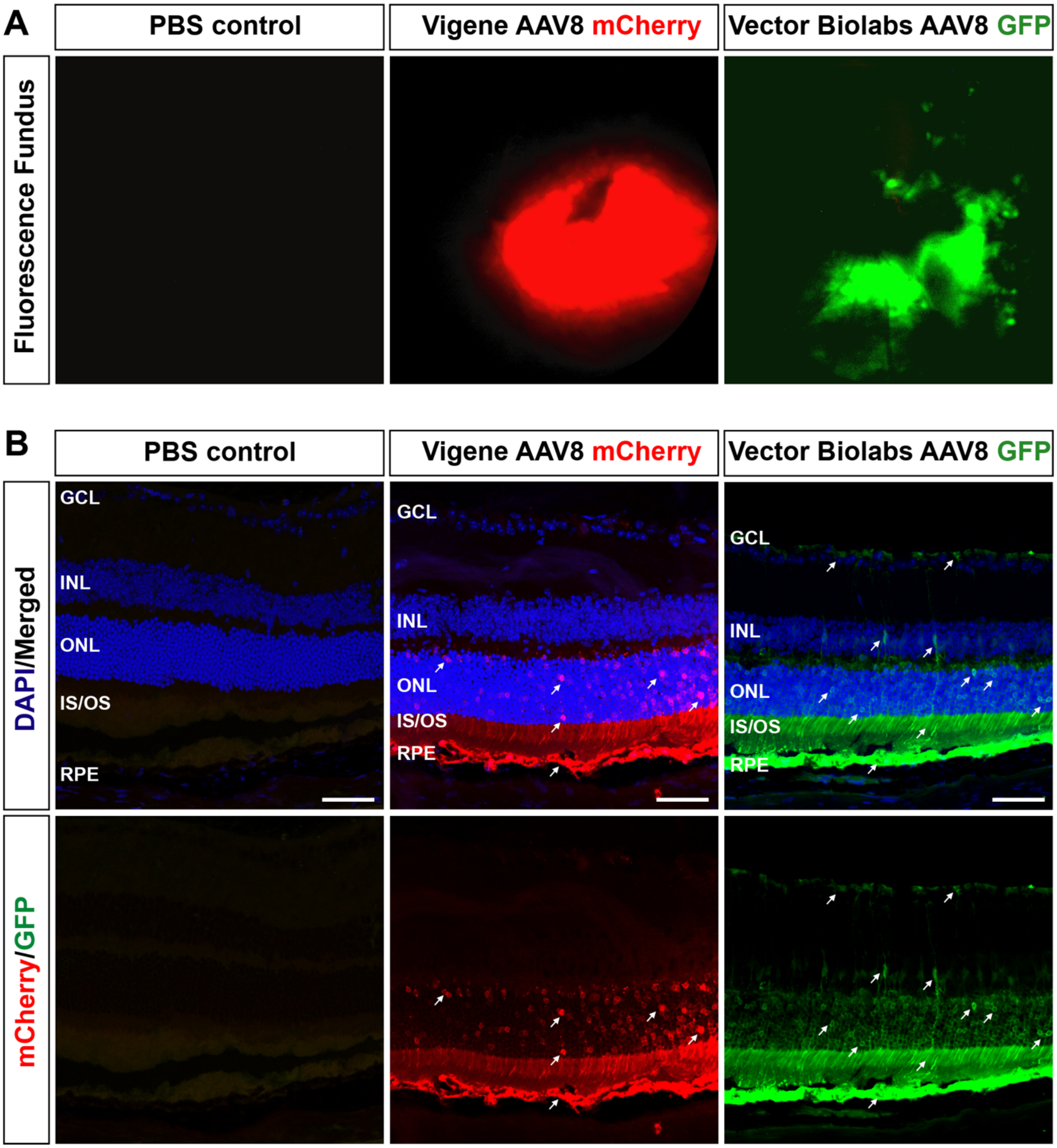

Figure S4. Comparison of AAV8 vectors from different suppliers. Three weeks after intravitreal injection of 1.9 x 10^7 GC of viral particles, AAV8 transduction (mCherry, red, Vigene or GFP, green, Vector Biolabs) was observed. (**A**) Fluorescence fundoscopy showing in vivo mCherry expression and GFP expression in wide view image of fundus. (**B**) Localization of transduced cells is shown in retinal sections. The mCherry positive cells transduced by the Vigene AAV8 are localized to ONL, IS/OS and RPE, whereas the GFP positive cells transduced by the Vector Biolabs AAV8 are likewise detected in ONL, IS/OS and (prominently) RPE, but also some GCL and INL cells. GCL, ganglion cell layer; INL, inner nuclear layer; ONL, outer nuclear layer; RPE, retinal pigment epithelium. Scale bars = 100 μm.

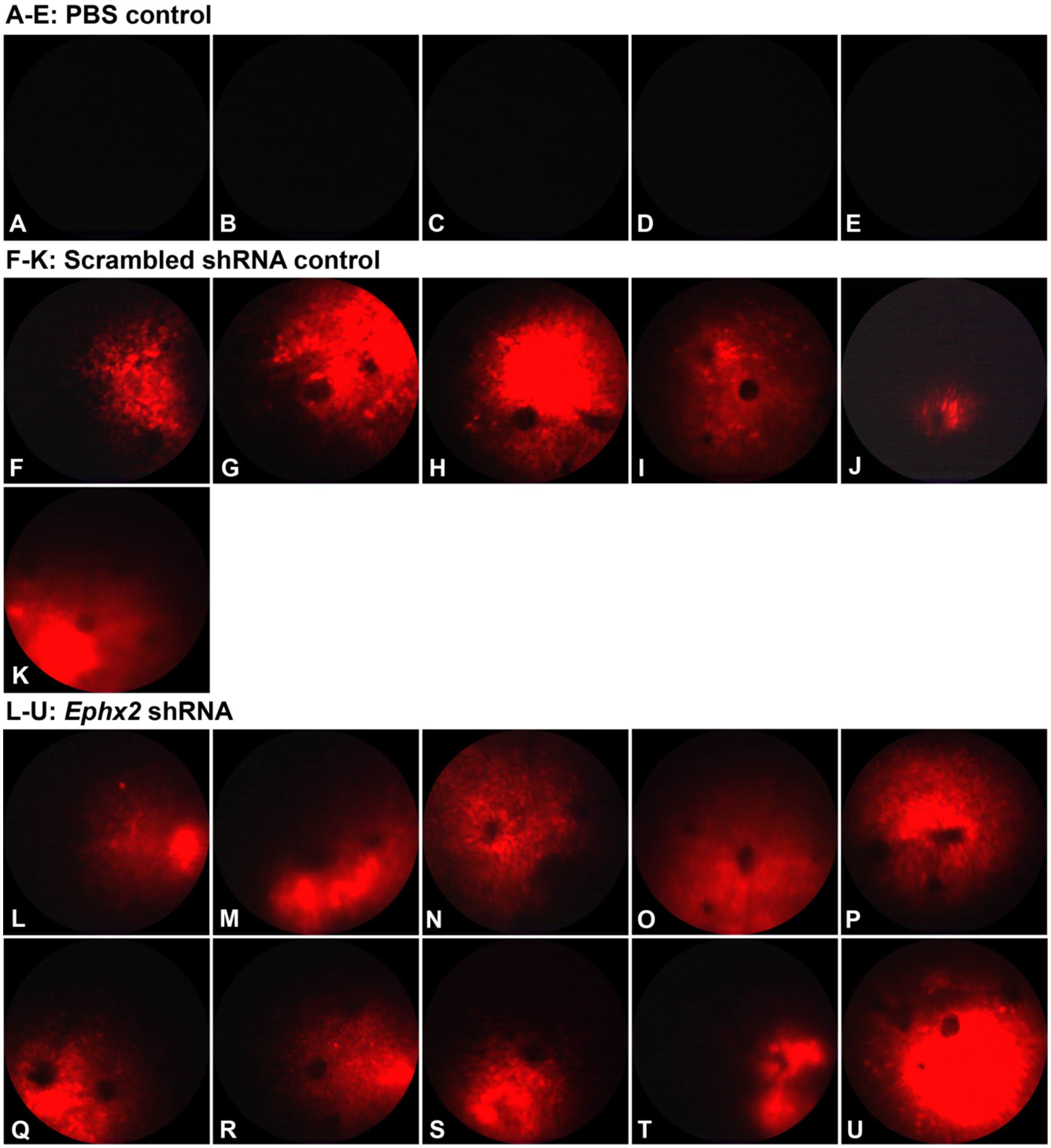

Figure S5. Fundus images of mCherry fluorescence in mice with L-CNV. mCherry fluorescence fundus images on day 21 (14 days post laser) of mouse eyes of PBS vehicle control (**A**-**E**), AAV8 viral vectors encoding scrambled shRNA control (**F**-**K**), and *Ephx2* shRNA (**L**-**U**).

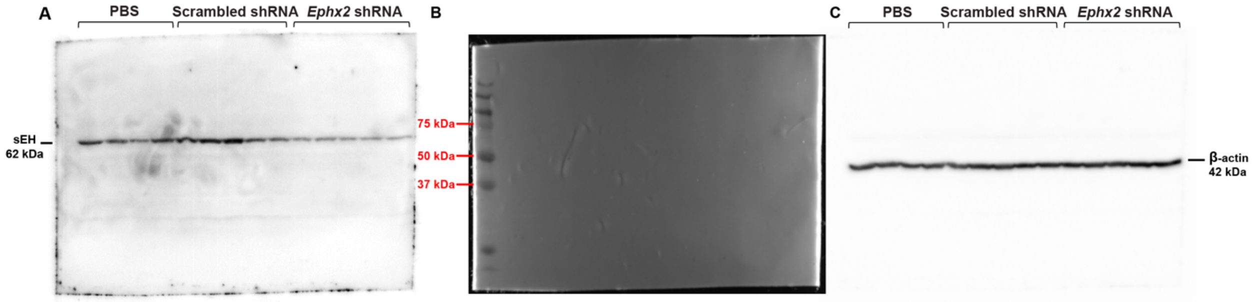

Figure S6. Full length immunoblot images from retina samples shown in Figure 7A. (**A**) Full length immunoblot image of sEH expression. (**B**) Molecular weight marker image. (**C**) Full length immunoblot image of β-actin expression.

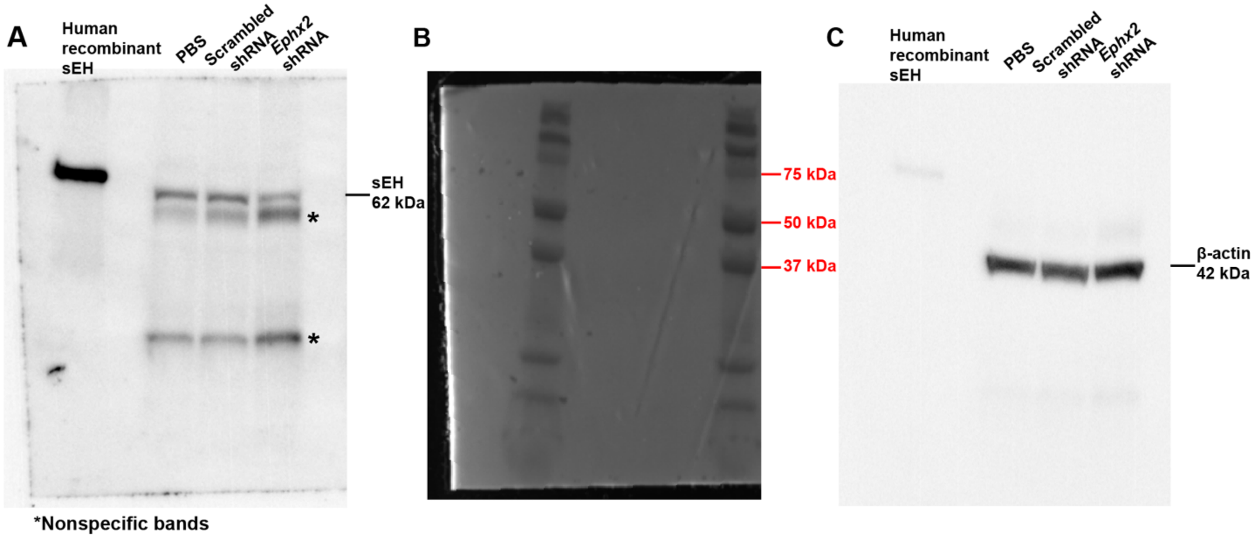

Figure S7. Full length immunoblot images from the pooled RPE/choroid samples shown in Figure 7B. (**A**) Full length immunoblot image of sEH expression. As positive control, purified human recombinant sEH was used. *Indicates nonspecific bands. (**B**) Molecular weight marker image. (**C**) Full length immunoblot image of β-actin expression.

Table S1. Antibody and lectin information for the human and mouse retina immunofluorescence and immunoblotting (IB; indicated in parentheses).

Antigen/Lectin	Catalog #	Supplier	Dilution ratio (1:N)	
			Human retina	Mouse retina
Soluble epoxide hydrolase (sEH)	A5	Santa Cruz	100	400, 1000 (IB)
Peanut agglutinin (PNA)	L6135	Sigma	250	N/A
Wheat germ agglutinin (WGA)	W32466	Thermo Fisher	250	N/A
Retinal pigment epithelium-specific 65 (RPE65)	ab231782	Abcam	400	500
Rhodopsin	ab3424	Abcam	N/A	300
Cone arrestin	AB15282	Millipore	N/A	500
Griffonia simplicifolia isolectin B4 (GS-IB4)	I21411	Thermo Fisher	N/A	250
β-actin	AC-49	Sigma-Aldrich	N/A	5000 (IB)

Table S2. TaqMan gene expression assays (Thermo) used in this study.

Target	Assay ID
Ccl2	Mm00441242_m1
Ephx2	Mm01313813_m1
Hprt	Mm03024075_m1
Icam1	Mm00516023_m1
Il1b	Mm00434228_m1
Il6	Mm00446190_m1
Tbp	Mm01277042_m1
Tnfa	Mm00443258_m1
Vegfc	Mm00437310_m1
